# Repeated and progressive rhabdomyolysis due to a novel carnitine palmitoyltransferase II gene variant in an adult male: A case report

**DOI:** 10.1097/MD.0000000000018143

**Published:** 2019-11-27

**Authors:** Lina Shao, Chunya Liu, Liyuan Xu, Rizhen Yu, Yiwen Li, Maosheng Chen, Qiang He

**Affiliations:** a Department of Nephrology, Zhejiang Provincial People's Hospital; b People's Hospital of Hangzhou Medical College, Hangzhou; c Jiangshan County People's Hospital, Quzhou, China.

**Keywords:** acute kidney injury, carnitine palmitoyltransferase II (CPT II), mutation, rhabdomyolysis

## Abstract

**Introduction:**

The occurrence of repeated and progressive rhabdomyolysis is rare in clinical settings, particularly in adults. The pathogenesis of rhabdomyolysis is often overlooked due to its rapid recovery. Carnitine palmitoyltransferase (CPT) II deficiency could be a rare etiology of repetitive nontraumatic rhabdomyolysis, and several mutations of *CPT II* have been reported.

**Patient concerns:**

A 41-year-old man presented with high fever, general malaise, myalgia, dyspnea, and dark-colored urine, and then progressed to anuria. In the past 15 years, he experienced dark-colored urine twice due to exercise and high fever. Physical examination revealed oliguria, suppurated tonsils, poor hemoglobin saturation, alert consciousness, normal neurological signs and reflexes, hypertension, and tachypnea. Laboratory investigations showed positive test results for inflammation, high serum myogenic enzyme levels, and evidence of acute kidney injury (AKI).

**Diagnoses:**

Investigations revealed an extremely high serum myogenic enzyme levels and impaired renal function with serum creatinine level of 510 μmol/L, consistent with the diagnosis of rhabdomyolysis, AKI stage 3, and acute respiratory distress syndrome. High levels of acylcarnitine in the serum confirmed the diagnosis of CPT II deficiency. In addition, whole exome sequencing (WES) was conducted in the patient and his mother.

**Interventions:**

Intubation, ventilator support, and hemodialysis were the major therapeutic interventions at the peak of disease progression. He was then administered valsartan tablets at a dosage of 80 mg per day and L-carnitine supplements.

**Outcomes:**

WES conducted in the patient and his mother revealed 2 novel mutations of *CPT II* (c.482G>A and c.1493G>T) in this patient. The patient recovered from the severe AKI but the renal function remained impaired at chronic kidney disease stage 3a.

**Conclusion:**

Thus, gene examination can help to understand the etiology of repetitive nontraumatic rhabdomyolysis. Accurate diagnosis can be beneficial for providing an individualized treatment for patients with repeated and progressive rhabdomyolysis.

## Abstract 2

Supplemental Digital Content is available in the text

## 1 Introduction

The carnitine palmitoyltransferase (CPT) system consists of 2 enzymes, CPT I and II; these function in the transport of long-chain fatty acids into the mitochondrial compartment. The enzymes are located in the outer (CPT I) and inner mitochondrial membrane (CPT II). The 3 phenotypes of CPT II deficiency are as follows: lethal neonatal, severe infantile hepatocardiomuscular, and mild myopathic forms.^[1]^ The clinical manifestations include muscle weakness, myalgia, pain, and rhabdomyolysis with or without renal failure. Trigger factors include prolonged exercise, fasting, fever, and exposure to cold.^[2]^ In this report, we describe a case of CPT II deficiency, caused by 2 novel mutations, that leads to repeated and progressive rhabdomyolysis with severe acute kidney injury (AKI).

## 2 Case presentation

**Figure 1:**
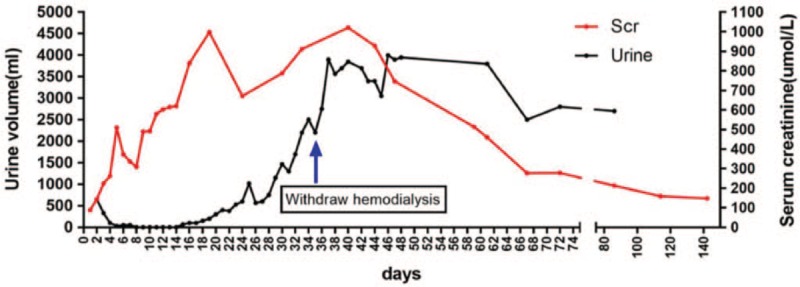
Change in urine and Scr levels during follow-up. Scr = serum creatine.

A 41-year-old man was transferred to the intensive care unit of a local hospital (Quzhou, China) because of severe rhabdomyolysis with AKI and acute respiratory distress syndrome (ARDS), on October 28, 2018. Twenty years ago, he had dark-colored urine after exercise. Approximately 15 years ago, he had the same symptom after severe cold with high fever; he was diagnosed with rhabdomyolysis, from which he recovered after general treatment. In the present condition, the patient reported of high fever, general malaise, myalgia, dyspnea, and dark-colored urine, and then progressed to anuria. An annual physical examination of the patient revealed no abnormal renal function. He never smoked and was an occasional drinker. He belonged to the ethnic Han community and had no prior family history of this disease. Physical examination on admission revealed oliguria, suppurated tonsils, poor hemoglobin saturation, alert consciousness, normal neurological signs and reflexes, arterial blood pressure of 165/95 mm Hg, and heart rate of 112 bpm. The initial laboratory investigations showed positive test results for inflammation (circulating leukocytes, 32.3 × 10^9^/L; C-reactive protein, 58.5 mg/L), high serum myogenic enzyme levels (aspartate aminotransferase, alanine aminotransferase, lactic dehydrogenase, and creatine kinase were 2 420, 623, 1 531, and 136 365 IU/L, respectively), and evidence of AKI at 48 h (proteinuria, 3+; urine occult blood test, 3+; blood urea nitrogen, 4.59–23.78 mmol/L; serum creatinine, 88.3–510 μmol/L). The serological test results for cytomegalovirus, Epstein–Barr virus, anti-glomerular basement membrane antibody, antinuclear autoantibodies, and other autoantibodies were negative. He was diagnosed with rhabdomyolysis with AKI and ARDS. The patient recovered successfully from ARDS with intubation and ventilator support, as well as continuous renal replacement therapy (CRRT); he was then removed from hemodialysis on day 48 of illness. Although he had normal 24-hour urine volumes, his renal function recovered gradually with abnormal serum creatinine during the 3-month follow-up period (Fig. 1).

Repetitive rhabdomyolysis following fever or exercise prompted us to investigate genetic and metabolic disorders. The blood carnitine level was detected by high performance liquid chromatography-tandem mass spectrometry and whole exome sequencing after obtaining a signed consent form.

**Figure 2:**
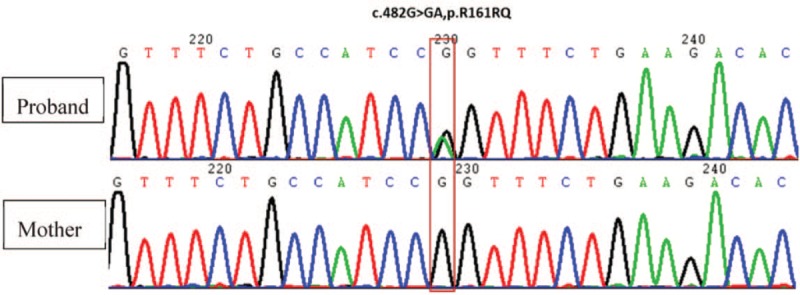
Schematic diagram representing the CPT II gene variation of the proband and his mother, based on Sanger sequencing results. CPT = carnitine palmitoyltransferase.

Adiacyl carnitine [C6DC] and 3-hydroxyoctadecyl carnitine [C18:1OH] were twice the normal upper limit (see Table, Supplemental Content, which illustrates the increased level of blood carnitine). The level of other carnitines was marginally higher than the normal upper limit. The abnormal carnitine levels showed carnitine metabolism dysfunction. There were 2 novel heterozygous and relatively rare mutation sites of *CPT II* (c.482G>A and c.1493G>T) (Fig. 2).

The missense mutation c.482G>A leads to an amino acid change of p.Arg161Gln. Its frequency is <1%‰ in the Genome Aggregation Database (gnomAD) and 0.2‰ in the general East Asian population. This variant has not been included in the 1000-genome project (genomic data for 2504 individuals from 5 populations), Exome Aggregation Consortium (ExAC) Exome Sequencing Project v. 6500 (ESP6500), Taiwan 500, CG69 (69 individuals with complete genomes), and local databases. The variant c.482G>A has not been reported. Neither the Human Gene Mutation Database (HGMD) nor ClinVar database (public archive of relationships among sequence variation and human phenotype) includes the mutation. The functional prediction showed the variant to be pathogenic. The predicted REVEL (Rare Exome Variant Ensemble Learner) and ClinPred (Prediction Tool to Identify Disease-Relevant Nonsynonymous Single-Nucleotide Variants) were 0.746 and 0.786, respectively.

The mutation c.1493G>T leads to an amino acid change of p.Arg498Leu. Its frequency in the general population is <1‰ in the ExAC and gnomAD databases and 0 in East Asian population. The variant is not included in the 1000 genome, ESP6500, Taiwan 500, cg69, and local databases. The variant c.1493G>T has not been reported. Neither the HGMD nor ClinVar database includes the mutation. The functional prediction showed it to be pathogenic; the predicted REVEL and ClinPred were 0.978 and 0.999, respectively.

The mutation c.1493G>T was verified using maternal peripheral blood. However, as paternal peripheral blood was not collected, the variation source of c.482G>A could not be determined. Furthermore, a genetic test related to the clinical phenotype was conducted; we did not identify any more gene mutations in the patient. Through bioinformatics analysis, data filtering, and comprehensive analysis, no pathogenic variation was found in the 59 genes recommended by the American College of Medical Genetics and Genomics Guidelines.

At 4 weeks after recovery, his urine output was almost normal. The blood creatinine kinase activity returned to the normal range without any muscle pain symptom. However, the patient refused renal biopsy. Even after >140 days of follow up, his renal function was abnormal, and was diagnosed as chronic kidney disease stage 3a with persistent microalbuminuria. He was then administered valsartan tablets at a dosage of 80 mg per day and L-carnitine supplements as the main course of treatment.

The research was approved by the ethics committee of Zhejiang Provincial People's Hospital. Patient has provided informed consent for publication of the case.

## 3 Discussion and conclusion

CPT II is a homotetramer 21 and anchors to the mitochondrial membrane with codon 464 through 496. Mutations leading to CPT II deficiency are located throughout the coding region. A mutation of serine at codon 113 to leucine (338C3T; S113L), which is the most frequent, has been reported to be closely related to adult-type rhabdomyolysis. Forty CPT II mutations distributed throughout the coding sequence of the gene have been reported. Thirty of the 40 mutations are missense mutations.^[1]^ Whole exome sequencing revealed 2 novel site mutations in CPT II – c.482G>A and c.1493G>T; neither has been reported. These mutations can lead to 2 amino acid changes that could cause CPT-II deficiency. Accumulation of long-chain acylcarnitines is a diagnostic marker of CPT-II deficiency.^[3]^ Our results showed a high level of long-chain acylcarnitines in patient's blood, due to CPT-II deficiency. We did not analyze CPT II expression in the patient because a previous study demonstrated that CPT II showed the same expression level in patients with genetically proven CPT II deficiency and normal control by immunohistochemical and western blot analyses of muscle biopsies. The CPT II-deficient enzyme activity and protein content were not reduced, but rather abnormally inhibited when fatty acid metabolism was stressed.^[4]^ The thermolability of the mutant enzyme can be attributed to muscle CPT II deficiency during prolonged exercise, infection, and exposure to cold.^[5]^ Typical symptoms, long-chain acylcarnitine accumulation, and genetic mutation confirmation of CPT II are essential for rhabdomyolysis diagnosis.

Positive urine protein and low urine specific gravity with abnormal serum creatinine suggested the persistence of renal damage in our patient. Dense cellular infiltration in the interstitium, with acute necrosis of the proximal tubule and intraluminal myoglobin casts in electron microscopic examination, has been reported in 1 case.^[6]^ These reported histopathologic findings strongly suggest tubulotoxicity of the myoglobin casts and resultant AKI. As our patient progressed to chronic kidney disease (CKD) with abnormal serum creatinine and persistent microalbuminuria, renal biopsy was recommended to assess the renal histopathological changes.

A genetic analysis should be recommended to adult patients with recurrent rhabdomyolysis, although most reported cases are of infants. Consequently, more genetic mutations can be detected and the mechanism can be studied further. With the development of gene editing technology, these mutation-related diseases can be cured.^[7,8]^ Based on the genetic analysis, accurate diagnosis helped guide this patient to effective prevention, such as avoiding strenuous activities and preventing respiratory infections. Long-term dietary therapy is also recommended, which is aimed at preventing any period of fasting. Restriction of long-chain fat intake along with medium-chain triglyceride supplementation is recommended.^[1]^ Some drugs, such as diazepam and ibuprofen, which might trigger attacks of rhabdomyolysis in CPT II-deficient patients, should be avoided. In conclusion, we report a novel mutation of CPT II in an adult man who presented repeated rhabdomyolysis with AKI and progressed to CKD. The discovery of novel mutations expands the information available on CPT II and will facilitate further study of its underlying mechanism.

## Acknowledgments

We would like to thank our colleagues at Zhejiang Provincial People's Hospital for their valuable contributions to this work.

## Author contributions

**Data curation:** Lina Shao, Chunya Liu, Liyuan Xu, Rizhen Yu.

**Funding acquisition:** Lina Shao, Qiang He.

**Investigation:** Lina Shao, Chunya Liu, Liyuan Xu, Maosheng Chen.

**Resources:** Yiwen Li.

**Supervision:** Yiwen Li, Qiang He.

**Writing – original draft:** Lina Shao.

**Writing – review & editing:** Rizhen Yu, Maosheng Chen, Qiang He.

Lina Shao: 0000-0002-6807-9320.

## Supplementary Material

Supplemental Digital Content
