# Supplementary material for: Repeated and progressive rhabdomyolysis due to a novel carnitine palmitoyltransferase II gene variant in an adult male: A case report
Source: Medicine (Baltimore). 2019 Nov 27;98(48):e18143. doi: 10.1097/MD.0000000000018143 (PMC6890328; doi:10.1097/MD.0000000000018143)
Supplement: Supplemental Digital Content [file medi-98-e18143-s001.docx]

| Supplementary table：Determination of carnitine by high performance liquid chromatography-tandem mass spectrometry | | | |
| --- | --- | --- | --- |
| Testing Item | Result |  | Reference Interval |
| Acetyl-L-Carnitine[C2] | 29.29 | ↑ | 1.28-21.60 |
| Butyl carnitine[C4] | 0.80 | ↑ | 0.06-0.42 |
| adiacylcarnitine[C6DC] | 0.51 | ↑ | 0.00-0.15 |
| Dodecyl carnitine[C12] | 0.13 | ↑ | 0.01-0.10 |
| 3-hydroxycetyl carnitine[C16OH] | 0.05 | ↑ | 0.00-0.03 |
| Octadecyl carnitine[C18:1] | 1.99 | ↑ | 0.23-1.74 |
| 3-hydroxyoctadecyl carnitine[C18:1OH] | 0.03 | ↑ | 0.00-0.02 |
| Octadecyl carnitine[C18:2] | 1.18 | ↑ | 0.06-0.56 |
| Glutaryl carnitine[C5DC] | 0.22 | ↑ | 0.00-0.20 |
| Isoprenyl carnitine[C5:1] | 0.07 | ↑ | 0.00-0.06 |
|  |  |  |  |
